# Supplementary material for: Spatial single-cell multiomics reveals peripheral immune dysfunction in Parkinson’s and inflammatory bowel disease
Source: NPJ Parkinsons Dis. 2026 Jan 16;12:25. doi: 10.1038/s41531-025-01199-2 (PMC12847817; doi:10.1038/s41531-025-01199-2)
Supplement: Supplementary file 1 — ALL Bolen CosMx Sup Figures_NPJPD_093025 MLB [file 41531_2025_1199_MOESM1_ESM.pdf]

Supplemental Table 1. Colonic biopsy patient demographics and cognitive status. Please see Supplemental Table 3 for additional metadata.

	**PD**<br>**N= 12**	**IBD**<br>**N= 13**	**NHC**<br>**N= 8**
**Sex F/M**	2/10	6/7	4/4
**Race/Ethnicity**	12 White	13 White	8 White
**NSAID use /never use**	4/8	8/5	3/5
**Age**<br>Avg ± SD<br>Median (IQR)	 62.9 ± 8.4<br>63.5 (6.5)	 56.5 ± 8.0<br>57 (8)	 64.6 ± 12.5<br>64.5 (14)
**Disease Duration**<br>Avg ± SD<br>Median (IQR)	5.55 ± 4.2<br>4 (7)	N/A	N/A
**MoCA Score**<br>Avg ± SD<br>Median (IQR)	25.64 ± 3.9<br>26 (8)	N/A	N/A
**Schwab and England ADL Scale**<br>Avg ± SD<br>Median (IQR)	90.09 ± 7.7<br>90 (5)	N/A	N/A

MoCA (Montreal Cognitive Assessment) Score: score reflecting the degree of cognitive impairment and signs of early dementia (score>26=normal, score<26=cognitive impairment).
Schwab and England Activities of Daily Living Scale: scale reflecting an individual's ability to function in activities of daily living (100%=very independent, 50%=very dependent, 0%=bed-ridden).
IQR: Interquartile range

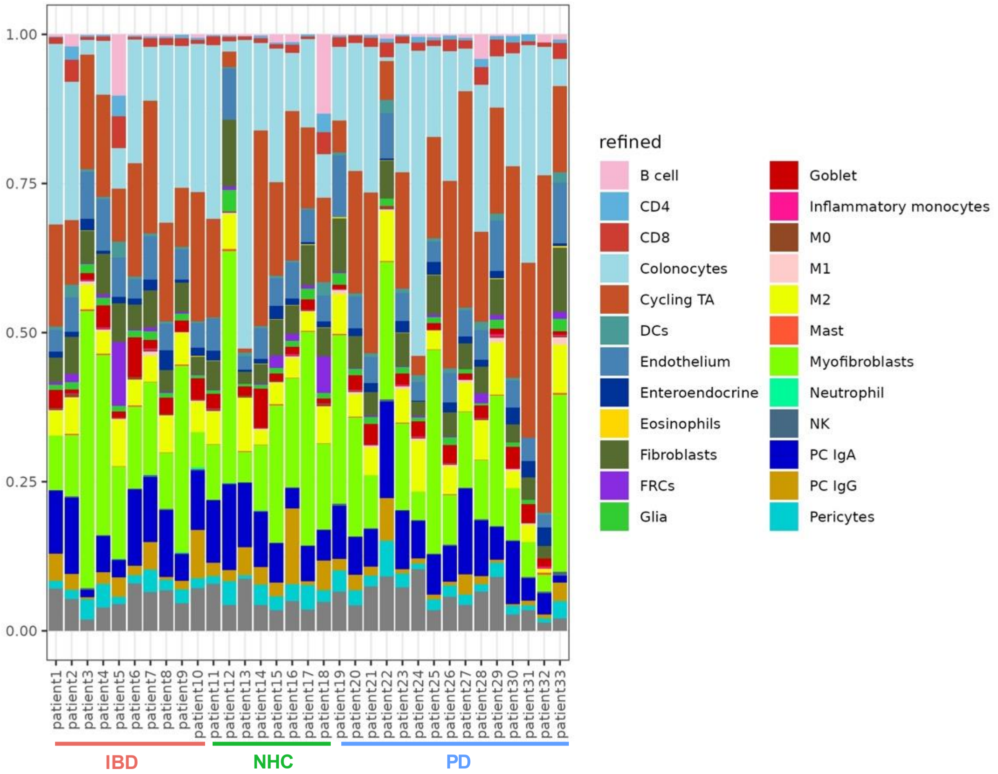

Supplemental Figure 1. Refined cell-type abundance from every participant. Patient anonymized ID (x axis) is used to identify the variance of annotated cell-types (y axis, 1.0= total cells accounted for) by participant.

Supplemental Table 2. CosMx SMI gene cluster identifiers. Cycling TA= cycling transit-amplifying cells. PC = plasma cells. NK = Natural killer cell. S2= fibroblast subtype. FRC = fibroblastic reticular cell. M0-2 = type of macrophage. DC= dendritic cell. These population markers were adapted from Garrido-Trigo et al., 2023.

Cell-type	Subpopulation Markers	Refined population markers
Epithelial cells	EPCAM, AQP8, BEST4, MUC2, OLFM4, PLCG2, TRPM5, ZG16	Colonocytes: AQP8, FABP1 and SLC26A2<br>Goblet cells: MUC2, TFF3 and SPINK4<br>Enteroendocrine: CHGA<br>Cycling TA: MKI67, TOP2A and PCNA
B cells	CD79A, BANK1, CD19, DERL3, MS4A1, MZB1	NA
Plasma cells	DERL3, MZB1, XBP1	PC IgA: +IgA and DERL3, MZB1, XBP1, -IgG<br>PC IgG: +IgG and DERL3, MZB1, XBP1, -IgA
T cells	CD3D, CD3E, CD3G, CD8A, FOXP3, GZMA, GZMB, IL17A, NKG7, TRBC1	CD8: CD8A, CD8B, GZMK, KLRB1, SPRY1 and IGTA1 and KLRG1<br>CD4: CD4, CCR7, LEF1, ANXA1, IL7R and GPR186<br>and SELL<br>NK: KLRF1, NCAM1, and lacks expression of CD3G, CD3D, CD8A and CD4
Stromal cells	ACTA2, ADAMDEC1, CHI3L1, COL3A1, NRXN1, PVALP, SOX6, VWF	S2: VSTM2, NPY and NRG1<br>Endothelium: VWF, PECAM1 and PLVAP<br>Fibroblasts: ADAMDEC1, CP, OGN, CCDC80 and GREM1<br>and FABP4<br>FRCs: CCL19 and CCL21<br>Glia: NRXN1, S100B and CDH19<br>Myofibroblast: SOSTDC1, ACTG2 and MYH11<br>Pericyte: NOTCH3 and RGS5
Myeloid cells	AIF1, C1QA, C1QB, CD14, CMTM2, FCGR3B, LYZ, MS4A2, TPSAB1, TPSAB2	M0: lack the expression of M2 markers and expression of CD68, C1QA, C1QB, SELENOP, LYZ, HLA-DPB1, HLA-DPA1, AIF1<br>M1: ACOD1,TNIP3, IL1B, INHBA, IL6, VCAN, CD300E, CXCL5, TNIP3, IL1B, INHBA,<br>IL6, VCAN, CD300E<br>M2: CD209, CD163L1 and FOLR2<br>Inflammatory monocytes: VCAN, CD300E, CD14, FCN1<br>and S100A9<br>Mast cells: LTC4S, TPSB2 and TPSAB1<br>Eosinophils: CLC, IL4 and IL13<br>Neutrophils: PROK2, CMTM2, CXCL8, FCRG3B, AQP9, S100A8 and S100A9<br>DCs: CD1C, TRL10 , TCTN3<br>CCL22 , CCL19, LAMP3

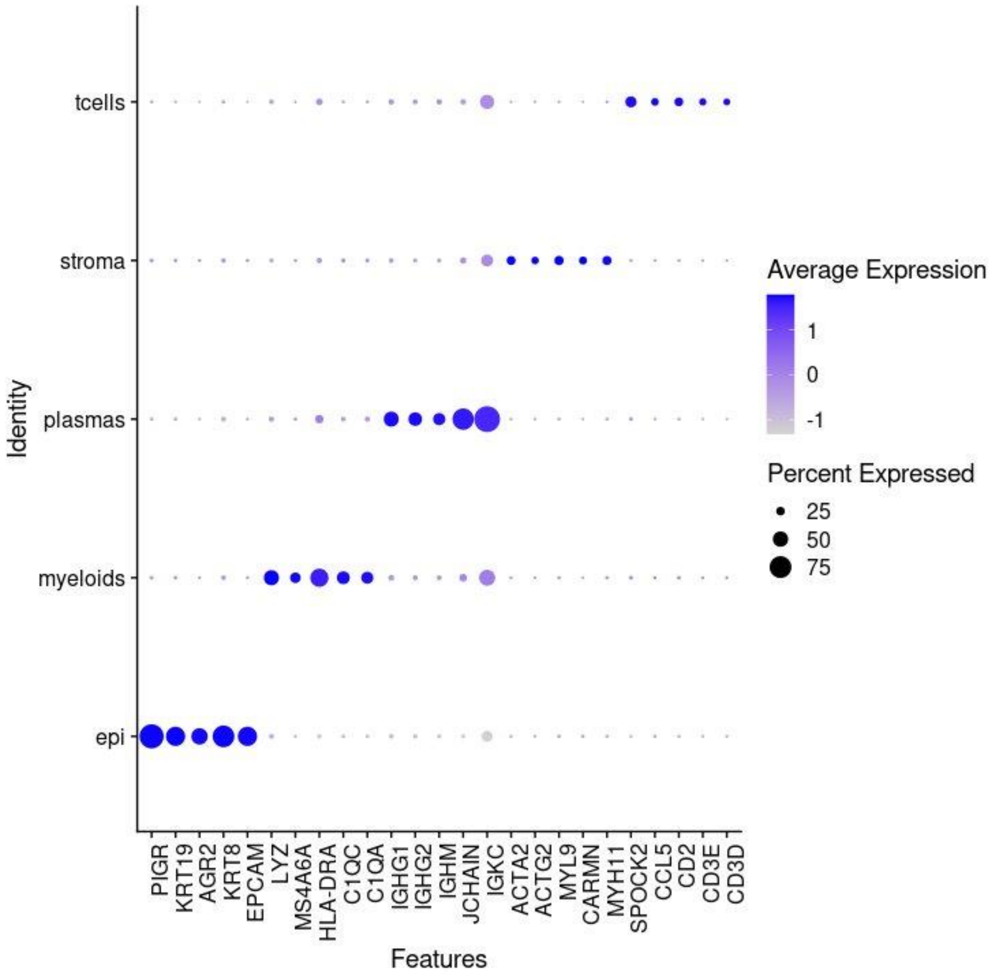

Supplemental Figure 2. CosMx SMI transcriptomics cell cluster identifiers. Dot plot of top gene markers used to cluster by annotated cell-type. The dot plot shows the size of the dot as the amount of expressing cells and mean expression levels (dot color). Total cells included: Epithelial= 29159, Myeloid cells = 5012, Plasma cells = 9446, Stroma cells = 27467 and T cells = 1988.

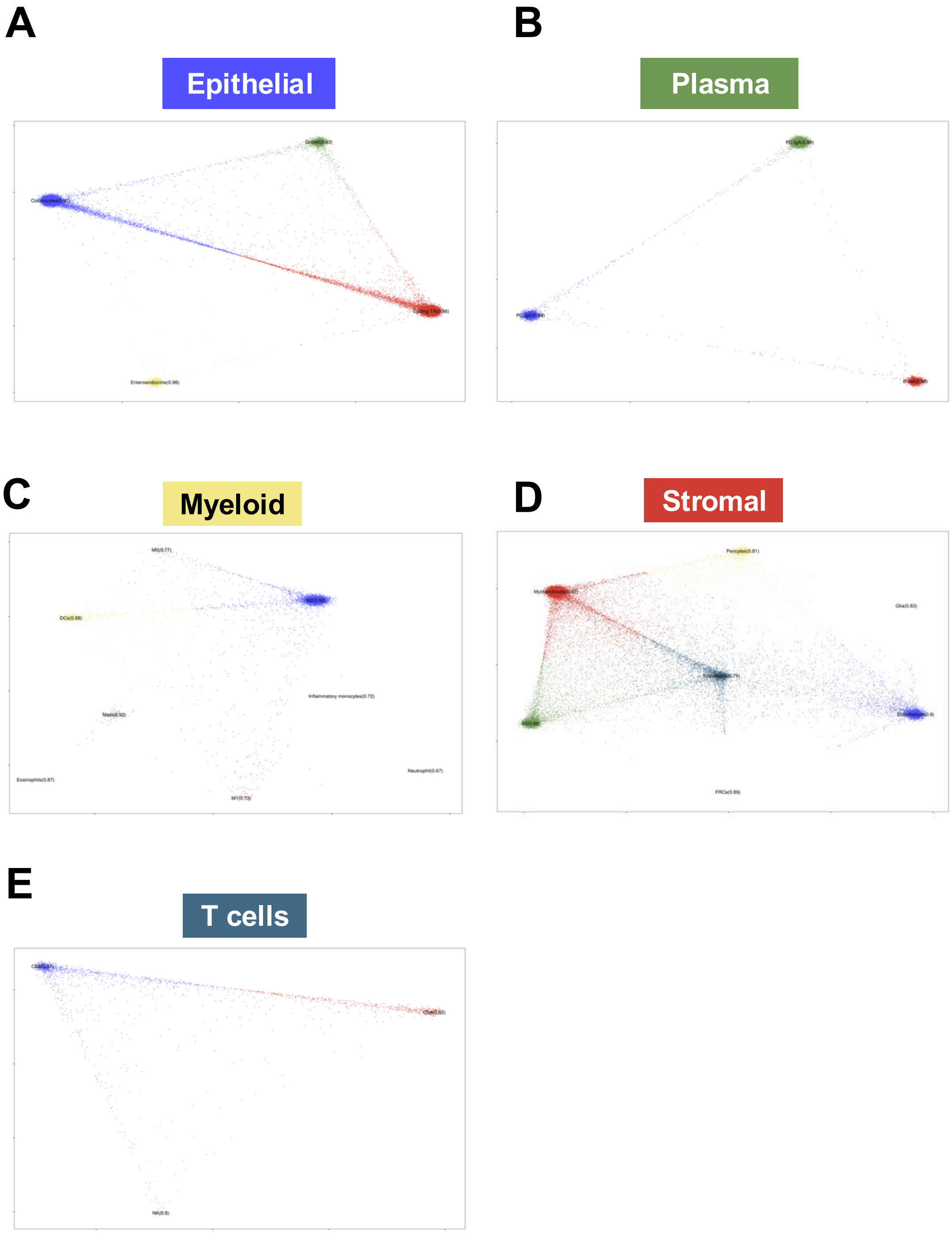

Supplemental Figure 3. CosMx SMI refined gene cluster flight-path analysis. Reduced dimensionality analysis representation of gene expression clustering by refined cell-type with all patients included not parsed by group.

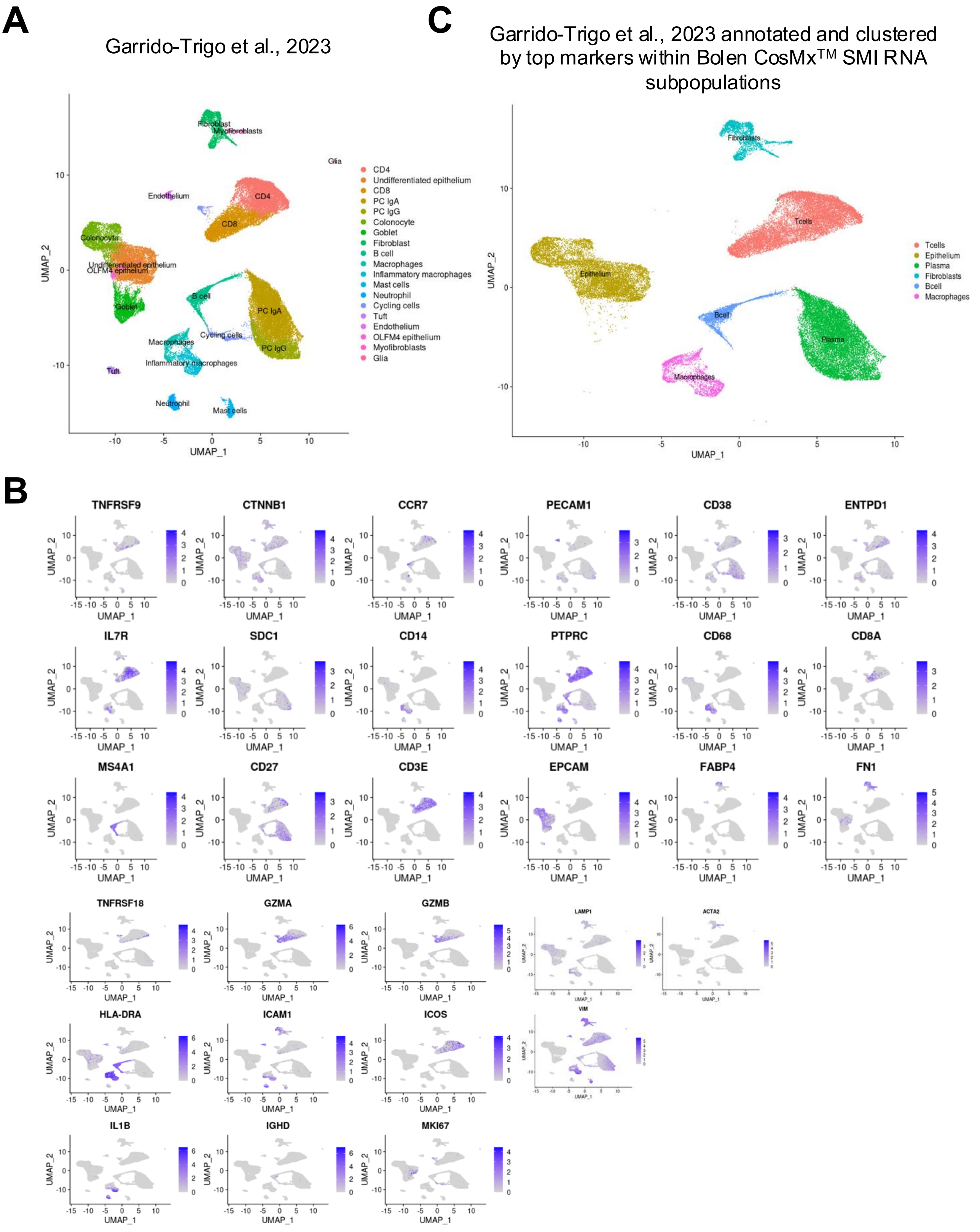

Supplemental Figure 4. Single-cell targeted proteomic analysis successfully integrates with scRNA-seq and depicts multimodal immune dysregulation across multiple biological outputs of human colonic biopsies. **A** UMAP representation of scRNA-seq of NHC human intestine biopsy from Garrido-Trigo et al., 2023 (n = 18 biopsy's). **B** Overlay of genes used within MaxFuse to allow for cross modal annotation of cells for CosMx SMI protein. **C** clustering of Garrido-Trigo et al dataset by gene markers from Bolen CosMx^{TM} SMI RNA dataset, then used to guide protein annotation (Fig 5).

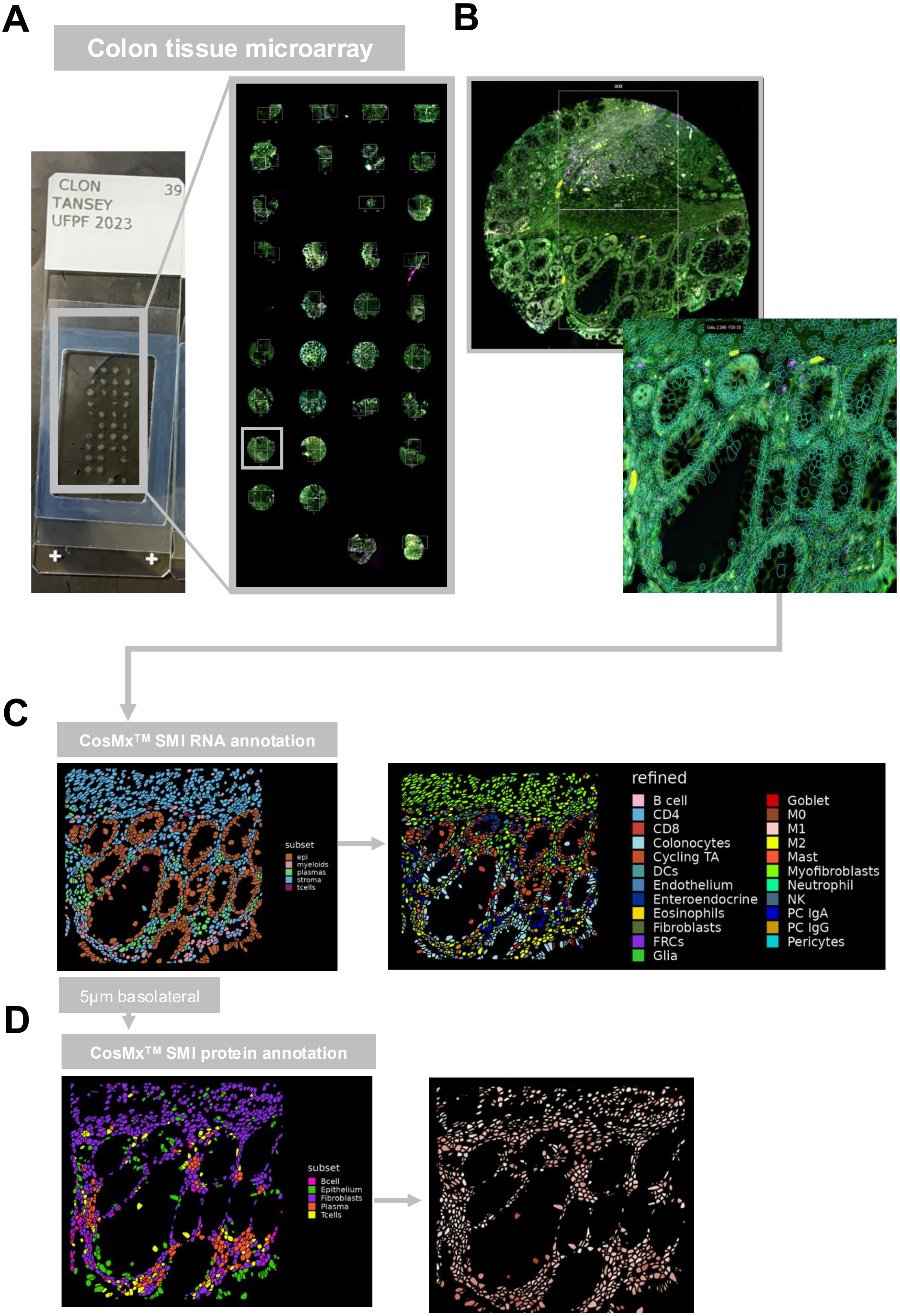

Supplemental Figure 5. Analysis workflow of CosMx^{TM} SMI RNA and protein analyses. **A** Tissue microarray of sigmoid colonic biopsies, all samples ran on the same slide with the same panel of antibodies. **B** CosMx^{TM} Spatial Molecular Imaging (SMI) visual representation of morphology markers, cell segmentation, cell annotation and protein expression. Morphology antibodies staining against nuclei (blue) and protein staining for cytokeratin (green), CD45 (magenta) and CD3 (yellow). **C** RNA cell annotation by subset and refined population **D** protein annotation by subpopulation and corresponding protein expression (1 example of 63).

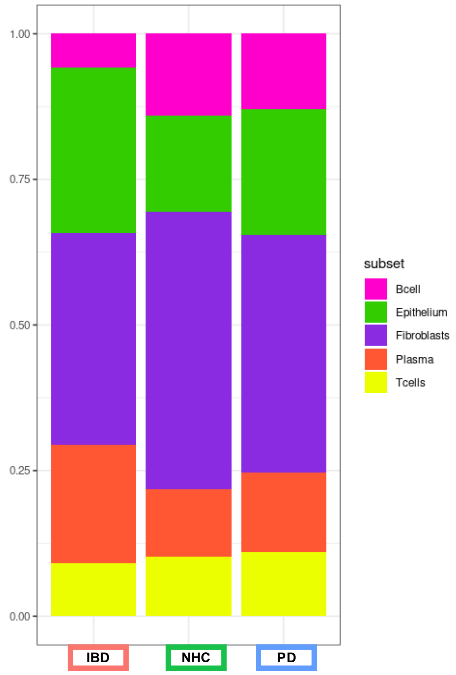

Supplemental Figure 6. Spatial molecular imaging (SMI) of directed proteome provides cell-type specificity and frequency at the level of subpopulations in sigmoid colon biopsies from those living with inflammatory bowel disease in remission (IBD) and Parkinson's disease (PD). Bar plot of SMI data are colored by group cluster identifier within each primary cluster. Each bar plot represents the proportion of cells in each subpopulation.

Supplemental Table 3. Colonic biopsy anonymized participant reported medication use. Yes = reported use, None = reported no use, IBD = inflammatory bowel disease, NHC = neurologically healthy control, PD = Parkinson's disease.

Group	Anti-TNF and/or direct anti-inflammatory	Dopamine therapy
IBD	None	None
IBD	Yes	None
IBD	Yes	None
IBD	Yes	None
IBD	Yes	None
IBD	Yes	None
IBD	Yes	None
IBD	Yes	None
IBD	Yes	None
IBD	Yes	None
IBD	Yes	None
IBD	Yes	None
IBD	Yes	None
NHC	None	None
NHC	None	None
NHC	Yes	None
NHC	Yes	None
NHC	None	None
NHC	Yes	None
NHC	None	None
NHC	Yes	None
PD	Yes	Yes
PD	Yes	Yes
PD	Yes	Yes
PD	None	Yes
PD	None	Yes
PD	Yes	Yes
PD	Yes	Yes
PD	Yes	Yes
PD	Yes	Yes
PD	None	Yes
PD	Yes	Yes
PD	Yes	Yes
